# Risk mapping and eco‐anthropogenic assessment of anthrax in the upper Zambezi basin

**DOI:** 10.1002/vms3.168

**Published:** 2019-03-28

**Authors:** Harvey K. Kamboyi, Michel de Garine‐Wichatitsky, Mudenda B. Hang'ombe, Musso Munyeme

**Affiliations:** ^1^ Department of Disease Control School of Veterinary Medicine The University of Zambia Lusaka Zambia; ^2^ Centre International de Recherche Agricole pour le Dévelopement UPR AGIRs Montpellier France; ^3^ Department of Biological Sciences RP‐PCP University of Zimbabwe Harare Zimbabwe; ^4^ Department of Para Clinical Studies School of Veterinary Medicine The University of Zambia Lusaka Zambia

**Keywords:** anthrax, eco‐anthropology, risk mapping, recurrence, Zambia

## Abstract

In Zambia, anthrax has emerged as a serious disease decimating humans, livestock and wildlife with devastating effects on eco‐tourism resulting in the destabilization of major pristine wildlife sanctuaries. Consequently, the thrust of this study was to establish the spatial distribution of anthrax and determine ecological drivers of its recurrence, maintenance and epidemiological linkage to anthropogenic activities. Environmental and biological samples were collected within the livestock production and conservation areas (*n *= 80). Each sample was serially tested for *Bacillus anthracis* positivity through blood agar culture and Gram stain technique, and then confirmation by multiplex polymerase chain reaction (MPCR). Questionnaires (*n *= 113) were conducted at independently distinct villages in terms of space and time. Most respondents showed that animals that died from anthrax were not properly disposed off. More likely than not, poverty being the main driver for anthrax carcass dressing and meat distribution contributed to environmental contamination with anthrax spores in areas where the animals subsequently died resulting in further environmental contamination, which is the major source of primary infection for livestock and wildlife. From the samples, 15 pure isolates of anthrax were obtained which were spatially distributed across four districts. Twelve, biologically plausible variables were found to be highly significant on multivariable logistic regression analysis model for questionnaires which included herd size (odds = 10.46; *P* = 0.005; CI 8.8–16), carcass disposal method (odds = 6.9; *P* = 0.001; CI = 3.4–9.8), access to veterinary services (odds = 10.87; *P* = 0.004; CI = 4.8–15.9) and management system (odds = 2.57; *P* = 0.001; CI = 1.3–7.5). In summary, the majority (78.7%) of anthrax outbreaks were observed in areas with low veterinary services (*χ* ^2^ = 8.6162, *P* = 0.013) within the newly created districts of Nalolo, Mwandi and Luampa.

## Introduction

Anthrax is an acute febrile disease of virtually all homoeothermic animals, including man, caused by a Gram positive, non‐motile, spore‐forming bacterium called *Bacillus anthracis*. When this multi‐host pathogen is excreted from an infected animal or when an opened carcass exposes the bacilli to free oxygen, they form spores that are resistant to extreme temperatures, chemical disinfectants and desiccation (The Merck Veterinary Manual, 1986). The spores can remain infective in soil for many years and during this time, they are a potential source of infection for grazing livestock, but generally do not represent a direct infection risk for humans (The Merck Veterinary Manual, 2014). Raw or poorly cooked contaminated meat is a source of infection for carnivores and omnivores. Anthrax resulting from contaminated meat consumption has been reported in pigs, dogs, cats, mink, wild carnivores and humans. Although the exact mode of transmission from cattle to each human is not usually identified, Munang'andu *et al*., in 2012 observed in Zambia that during outbreaks people involved in skinning infected carcasses, handling meat and processing skins and hides for making drums or stools were more susceptible to the disease than those who consumed cooked meat. Meat from animals that died on the floodplains was carried to upland areas to the owners for consumption or to be shared or sold.

In Zambia, anthrax has emerged as a serious ecological disease in livestock and wildlife with a devastating effect on the ecotourism and a potential threat on the socioeconomic viability of three pristine wildlife sanctuaries of the upper Zambezi basin, the Lower Zambezi and the Luangwa valleys. Although outbreaks of the disease have occasionally been reported from different parts of the country (Turnbull *et al*. 1991, 1999; Tuchili *et al*. 1993), the disease has increased to alarming levels in recent past (Siamudaala *et al*. 2006). The upper Zambezi basin has reached an endemic status of anthrax which means the area has an ecological threshold ideal for the appropriate mix of suitable parameters that favour the maintenance and transmission of anthrax pathogens to susceptible populations existing within the ecological habitat. For the period 1999 ‐ 2007 (Munang'andu *et al*. 2012) reported a total of 1216 bovine cases, 1790 human cases and a human case fatality rate of 4.63% (83/1790) in the Zambezi flood plain. In this study, human cases were highly correlated with cattle cases (*r = *0.94). According to the authors, the initiation of outbreaks of anthrax depends on interrelated factors which include specific properties of the bacterium, environmental factors and factors affecting the dissemination of the organism, animal densities and certain human activities. Therefore, the thrust of this study was to map and identify high risk areas and to carry out an eco‐anthropogenic assessment for anthrax in the upper Zambezi basin.

## Materials and methods

### Study area

The upper Zambezi basin also called the Barotse floodplain lies 14°19′–16°32′S and 23°15′–23°33′E and covering about 5,500 km^2^ in extent (IUCN, 2003). The study area includes part of the Kavango‐Zambezi Trans‐Frontier Conservation Area (KAZA TFCA) in Sioma Ngwezi.

**Figure 1:**
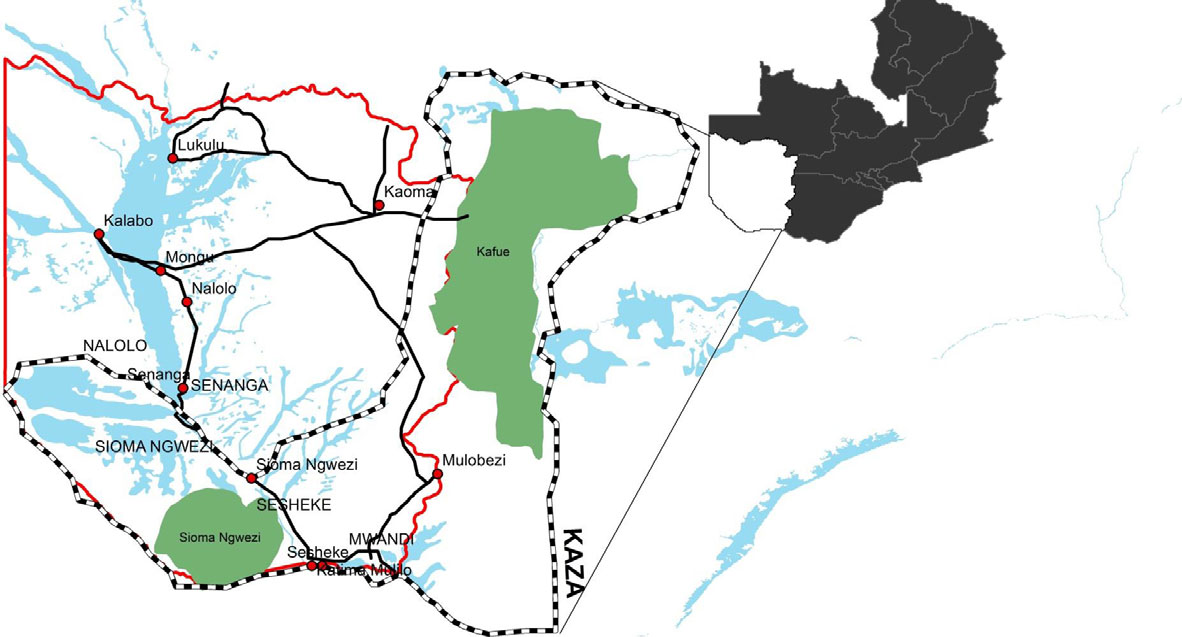
Map of western province of Zambia showing the study area with important physical features.

The maximum flooded area is estimated at 10 750 km^2^ (Welcomme 1985) when floods of all tributaries of the Zambezi river are taken into account (Fig. 1). The floodplain stretches from the confluence of the Lungwebungu River with the Zambezi River in the north extending southwards for a distance of 250 km until Ngonye falls (Fig. 1). Soils are composed of the Kalahari sands stretching several meters deep underlain by calcareous rocks (Munang'andu *et al*. 2012). Elsewhere calcareous soils have been associated with prolonged survival of anthrax spores (Hugh‐Jones & Hussaini 1975; New *et al*. 2002). The major human activities in the study area are traditional cattle farming, fishing and rice farming and to a lesser extent, maize farming in the upper lands. The cattle population in the study area is estimated at 400 000 as of 2014 according to the National Livestock Epidemiology and Information Centre (NALEIC) a unit of the Department of Veterinary Services mandated to coordinate surveillance and information processing activities. The floodplain contains a population of 225 000 people in an estimated 28 000 households.

### Study design

The research was cross‐sectional, employing opportunistic biological sampling of carcasses and/or their remains, active environmental sampling of soil and water points in villages suspected to have had any history of livestock anthrax cases. Pre‐tested structured questionnaires were utilized in data collection.

### Data collection and analysis

#### Questionnaires

A questionnaire survey was conducted during the data collection stage to determine the knowledge, attitudes, beliefs and opinions of the local people about anthrax that may help to explain the disease recurrence. A total of 113 questionnaires were distributed in villages within the study area that had had a history of the disease and those without any history. This ensured that there was an unbiased evaluation of the levels of awareness about anthrax. The target population included all cattle farmers in the upper Zambezi basin and the study population comprised simple randomly selected villages with cattle farmers. The sampling unit of interest was a village and the secondary sampling unit was a simple randomly selected individual cattle farmer in each of the selected villages. GPS coordinates were also entered for each completed questionnaire; the information therein was used for the mapping of past anthrax cases. STATA™ version 13 software was used for quantitative analysis. The outcome of interest was occurrence of an outbreak at village level to obtain frequencies, proportions, risk factor association using chi square test and multiple logistic regression reporting odd ratios to test for causal association.

#### Biological and environmental samples

The spatial distribution map was obtained from the compounded biological and environmental samples (*n *= 80) that were collected randomly in livestock production areas and also in areas that had been previously reported to have anthrax outbreaks. The area covered was 15.82073–17.199 latitude and 23.03045–24.52548 longitude. Biological samples comprised fresh carcass tissues and exudates as well as old bones and hides of animals presumed to have died of anthrax. The size of the sample ranged from the entire shaft of the long bone (femur/tibia), entire spleen to 2 cm diameter of dried hide. Environmental samples included soil, dung and water specimens collected from wallows and open meadows and also from carcass disposal sites. Mostly environmental specimens were composed of a heterogeneous mixture, including soil, animal faces from carcass and vegetation near disposal sites. Both biological and environmental samples were purposively and conveniently collected in areas where cattle anthrax cases had been reported. During collection of specimens, the areas were considered contaminated and appropriate safety precautions had to be taken. Global positioning system (GPS) coordinates were recorded for each of the samples was be collected. The samples were cultured on blood agar media for *Bacillus anthracis* colony characterization. The protocol followed was as that described in the OIE terrestrial manual version adopted by the world assembly of delegates of the OIE in May 2012. Suspected colonies were then subjected to Gram stain techniques for further diagnosis. Samples that tested positive to both culture and gram stain, had their deoxyribonucleic acid (DNA) extracted which was used for multiplex polymerase chain reaction (MPCR) for confirmation of *B. anthracis*.

## Results

### Questionnaire surveys and identified risk factors associated with anthrax

**Figure 2:**
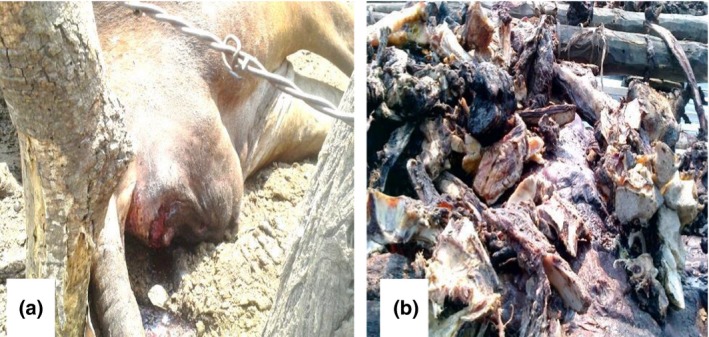
(a) A cow that died from anthrax with visible blood oozing from the anus and was heavily bloated. (b) Smoked beef from an anthrax carcass, Nakapungu village, −16.19932S 23.17062E Sioma Ngwezi district 2015.

A total of 113 households were interviewed and these formed the core for which identified risk factors associated with anthrax were generated. Variables considered to be significant on univariate analysis (Table 1) were assessed via a multivariable logistic regression model. All the main variables with their interaction variables were included as predictors in the model, as each variable or its interaction could affect the odds of an animal being positive for anthrax. All the variables and their interactions were previously analysed using contingency tables, and those that were statistically significant at 95% CI (*P* < 0.05) on univariate analyses were included in the full model. Twelve biologically plausible variables were found to significantly increase the risk of anthrax outbreaks on multivariable logistic regression analysis model (Table 2). The model was tested for the goodness of fit using the Hosmer–Lemeshow statistic. The same test was used to test the model's sensitivity and specificity at different probability cut points (Fig. 2).

**Table 1:** Risk factors for anthrax in traditional cattle in upper Zambezi basin (questionnaire survey, November 2014 to August 2015, *n *=* *113)

Risk factor	Levels	n	Percentage	No. of anthrax cases	Percentage of the anthrax cases
Herd size	0: ≤30	43	38.05	14	18.67
	1: 31–60	51	45.13	41	54.67
	2: 61–90	15	13.27	16	21.33
	3: ≥91	4	3.54	4	5.33
Management system	0: Transhumance	75	66.37	63	85.11
	1: Free range	38	33.63	12	14.89
Grazing distance	0: ≤5 km	40	35.21	11	14.89
	1: >5 km	73	64.79	64	85.11
Grazing duration	0: <1 month	38	33.8	11	14.89
	1: 1–2 months	6	5.63	3	4.26
	2: >2 months	68	60.56	61	80.85
Grazing months	0: April to Nov	62	54.93	53	70.21
	1: Aug to Dec	8	7.04	5	6.38
	2: Non‐specific	43	38.03	18	23.41
Year of observation	0: 2010 – 2014	88	77.46	64	85.11
	1: 2004 – 2009	25	22.54	11	14.89
Kraal mortality numbers	0: ≤ 10	75	66.67	49	65.96
	1: 11–50	35	31.25	24	31.91
	2: ≥ 50	3	2.08	2	2.13
Village mortality numbers	0: ≤ 10	39	43.66	41	55.32
	1: 11–50	27	23.94	24	31.91
	2: ≥ 50	37	32.39	10	12.77
Disease management	0: Treatment	74	65.38	49	65.96
	1: Do nothing	26	34.62	26	34.04
Carcass disposal	0: Dressed	104	92	69	91.49
	1: Disposed	9	8	6	8.51
Vet‐services	0: None	44	39.44	37	48.94
	1: Low‐Med	32	28.17	22	29.79
	2: High	37	32.39	16	21.77

**Table 2:** Final multivariate logistic regression analysis of risk factors for anthrax outbreaks in cattle

Risk factor	SE	Odds	*P*	95% CI
Herd size	0.1	10.46	0.005	8.8–16
Management system	0.05	2.57	0.001	1.3–7.5
Grazing distance	1	14.99	0.0001	7.3–24.4
Grazing duration	1.9	14.84	0.0001	5.4–17.58
Grazing months	2.1	4.08	0.001	2.8–7.3
Year of observation	0.8	3.1	0.003	1.5–4.9
Kraal mortality	2.57	17.96	0.001	15.4–27.9
Village	1.36	5.69	0.0001	3.6–7.2
Disease management	0.42	4.32	0.02	2.2–6.2
Carcass disposal	1.23	6.9	0.001	3.4–9.8
Vet‐services	0.6	10.87	0.004	4.8–15.9

Not significant (*P* > 0.05), Likelihood ratio = 45.91.

Number of observations in the model = 113.

Hosmer–Lemeshow chi^2^ (8) = 6.71, Prob > chi^2^ = 0.938.

### Biological and environmental samples

**Figure 3:**
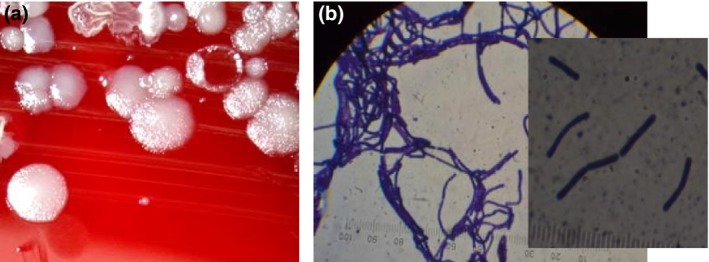
(a) Greyish white non‐haemolytic colonies of *Bacillus anthracis* on blood agar and (b) Gram positive B. anthracis rods at ×100.

The 80 samples that were collected and cultured on blood agar, 36 grew characteristic *B. anthracis* colonies. The colonies were grey‐white to white in colour and measuring 0.3–0.5 cm in diameter and non‐haemolytic (Fig. 3a). Thirty‐three (33) of the colonies tested positive on gram stain (Fig. 3b).

### Multiplex polymerase chain reaction (MPCR)

Suspected samples that tested positive on both culture and gram stain (*n* = 33) as shown in Fig. 3 were subjected to MPCR for confirmation. MPCR was used to amplify DNA, and in the case of *B. anthracis* it was used to amplify four (4) specific genes namely:

1. *B. anthracis* specific fragment (BA),
2. Toxin gene fragment – Protective antigen (PA)
3. Capsule gene fragment (CAP)
4. Bacteria‐specific fragment (16S)

**Figure 4:**
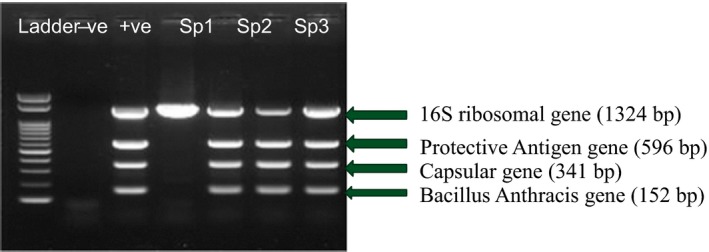
Multiplex PCR products, positive sample showing characteristic separation into four clear gene bands at the same level as the positive control. Negative samples had less or more than four unclear gene bands at different levels.

Fifteen pure isolates were confirmed positive on MPCR (Fig. 4).

### Mapping of anthrax positive samples

**Figure 5:**
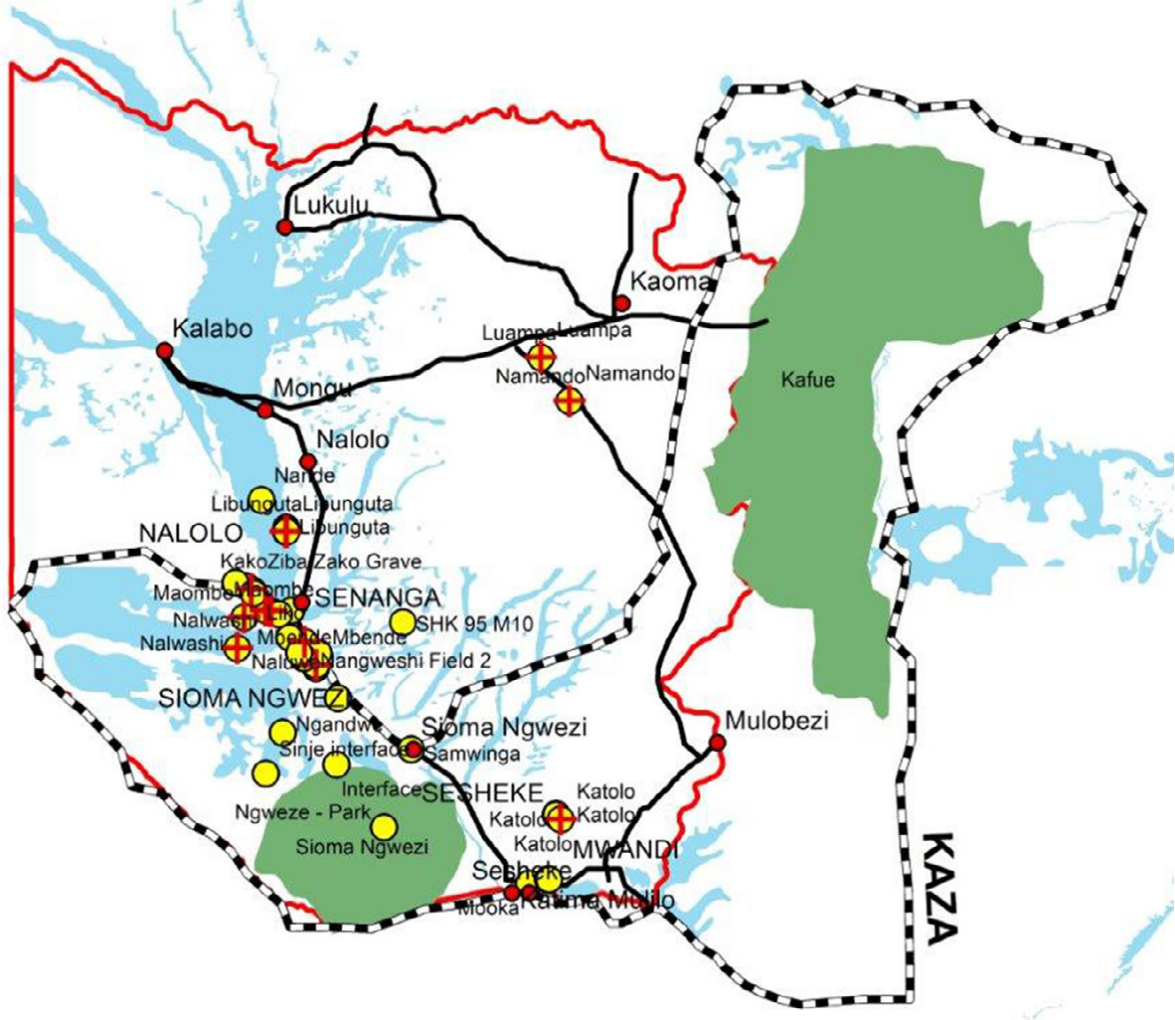
Spatial distribution of anthrax at village level. The yellow dots indicate the coverage of the sampling exercise (both within and outside the KAZA‐TFCA), whereas the red crosses represent samples that tested positive on PCR (refer to Table 3).

The raw GIS data obtained during biological and environmental sampling and imported into ArcGIS for geoprocessing generated spatial distribution maps (Fig. 5). The yellow dots indicate the coverage of the sampling exercise (both within and outside the KAZA‐TFCA) whereas the red crosses represented samples that tested positive on PCR (Fig. 5).

## Discussion

In the most affected areas, a perception has seemingly developed among the inhabitants that the yearly outbreaks are not uncommon. This was very clear from the answers that were provided from the questionnaires. The major method of disposal of anthrax carcasses was consumption (Fig. 2) as indicated by 92% of the participants. This practice was observed across the entire study area. It is assumed anthrax is not a fatal disease unlike malaria due to lower levels of public health significance posed by the disease. Given this background, the inhabitants readily open up anthrax carcasses (Fig. 2), which results in the sporulation of the bacteria and environmental contamination (Siamudaala *et al*. 2005), although they may not aware of this epidemiological consequence of their action. This scenario is worsened by the lack of veterinary intervention, as a direct association was found between lack of veterinary services and frequency of anthrax outbreaks. Areas that received veterinary intervention and good extension services were less likely to experience frequent anthrax outbreaks. Additionally, in areas where such services were not available, neither systemic disease surveillance nor monitoring programs are in place, 78.7% (37/47) (*χ* ^2^ = 8.6162, *P* = 0.013) of anthrax cases emanated from there. During severe outbreaks, the course of the disease is dependent on ‘self‐limiting factors’ such as reduced cattle densities during outbreaks (De Vos 1990) whilst the people don't dispose of the carcasses. The existing poor infrastructure, starting from the road networks, lack of funding to most rural veterinary and health outposts mean authorities in these areas have no planned activities to cover issues like anthrax despite their serious nature (Munang'andu *et al*. 2012). Information from veterinarians in the study area indicated that most of the resources and activities are directed towards the prevention and control of foot and mouth disease (FMD) and contagious bovine pleural pneumonia (CBPP). Anthrax control and prevention is left to the cattle farmers even when it is not only a notifiable disease under the current statutes but also a zoonotic one. The CBPP and FMD surveillance system may only switch to focus on anthrax when tens or hundreds of cattle will have died, and the meat consumed by humans and domestic carnivores and the environment is contaminated with anthrax spores.

The first key finding from this study is that we have been able to map and identify high risk areas with regards to frequency of anthrax outbreaks in the upper Zambezi basin. Additionally, this study has elucidated the ecological (flooding and geology) and anthropogenic factors (carcass dressing) for anthrax occurrence and sustained outbreaks in the study area. Ecological factors that were significant and seen on the mapping data included elevation/altitude, with almost all the outbreaks occurring in the low‐lying areas and seldom in the higher areas. This link in ecological determinants is well elucidated when outbreaks are overlaid according to topography as reports of anthrax in both the epizootic and sporadic form in the upper Zambezi were all confined in the lower flood plains with none in the higher plateau. Figure 5 and Table 3 show that the majority of the anthrax isolates were distributed between −15.039 and −16.4523 latitudes and 23.039 to 24.52514 longitudes which are low veld areas. This finding was also observed by Munang'andu and co‐workers who attributed this observation partly to the alluvial soils of the plains (Munang'andu *et al*. 2012). From the final multivariate multiple logistic regression model, anthropological factors that were strongly associated with anthrax outbreaks included herd size, cattle management systems (especially transhumance animals that were grazed in the flood plains most times of the year). Other risk factors included modes of carcass disposal, veterinary services availability among many others. Another significant risk factor of anthrax outbreak was history of having had an animal that had previously died in the same kraal. Those kraals which reported animals having died before in the same kraals were almost 18 times more likely to experience another outbreak than those that did not have had a previous occurrence (odds ratio 17.96, 95% CI 15.4–27.9 with *P *<* *0.001).

**Table 3:** Biological and environmental sample that tested positive for *Bacillus anthracis*

SN	ID	District	Latitude (S)	Longitude (E)	Sample
1	Mbende	Sioma Ngwezi	−16.4523	23.40017	Soil
2	Mbende	Sioma Ngwezi	−16.4523	23.40017	Horn
3	Katolo	Mwandi	−17.1593	24.52506	Soil
4	Katolo	Mwandi	−17.1596	24.52514	Soil
5	Nambwele	Sioma Ngwezi	−16.20358	23.19485	Soil
6	Nangweshi Field 2	Sioma Ngwezi	−16.34428	23.34861	Soil
7	Nakapungu Kalongola 3	Sioma Ngwezi	−16.19932	23.17062	Ear
8	Nakapungu Kalongola 4	Sioma Ngwezi	−16.19932	23.17062	Spleen
9	Libunguta	Senanga	−15.83903	23.26246	Soil
10	Libunguta	Senanga	−15.83807	23.26484	Water
11	ZibaZako Grave	Nalolo	−16.10482	23.10377	Soil
12	Maombe	Nalolo	−16.23252	23.07052	Soil
13	Luampa	Kaoma	−15.039	24.434	Soil
14	Namando	Kaoma	−15.239	24.565	Ear
15	Nalwashi	Sioma Ngwezi	−16.374	23.039	Soil

Geographical location of the positive anthrax isolates based on molecular typing of environmentally collected samples has helped us to elucidate essential characteristics linked to this disease in Western Province of Zambia. Frequent outbreaks in the same areas and the type of anthropological activities were found to be directly correlated as generated from the final logistic regression model. These observations are congruent with those of Siamudaala *et al*. (2006), who found that dressing of anthrax carcasses by humans was more at play in the continuous outbreaks of anthrax in the Zambezi basin of Western Province than in the Luangwa basin. Similarly, Munang'andu and co‐workers attributed this observation to consumption of dead carcasses as a sequel of an anthrax outbreak (Munang'andu *et al*. 2012). From the multiple logistic regression analysis, transhumance grazing system was one of the significant risk factors of anthrax occurrence in the upper Zambezi basin. Farmers that practised the distant transhumance grazing system were approximately 15 times more likely to be affected by anthrax (odds ratio 14.99, 95% CI 7.3–24.4 with *P* < 0.001). Cattle owners with a herd size greater than 30 were 9 times more likely to practice transhumance grazing system (odds ratio 8.92, 95% CI 6.8–9.4 with *P* < 0.001). This explains why herd size is also a risk factor for anthrax outbreaks. Herdsmen practise a transhumance grazing system that brings cattle into the wetlands during the dry season. At the peak of the dry season cattle herds are concentrated around the lagoons and oxbow lakes within the flood plains, as the grass becomes overgrazed, cattle inhale and ingest the spores from the grass and in drinking water, respectively. Outbreaks start in the dry season extending until the onset of the rainy season. By the peak of the rainy season most animals will have been moved to the uplands. Hence, no outbreaks are reported during this period until the next dry season.

Complementary to ecological parameters, increase in the susceptible livestock population, transhumance grazing system, seasonal anthropogenic activities and pressure, poor public awareness and the general lack of a systematic intervention program have led to a cyclical trend in the recurrence of anthrax outbreaks on the upper Zambezi basin.

## Conclusion

Although anthrax is perceived as a potential weapon of bioterrorism in some countries in the world, it has ecologically emerged to be a significant public health threat in the Western Province of Zambia especially along the Zambezi basin. The endemic situation results from a mix of ecological and anthropogenic parameters such as the cyclical rainfall pattern, flooding, evaporation potential, temperature, the geology of the floodplains, with complimentary epidemiological factors like the increase in cattle and human populations, transhumance grazing system, and anthropogenic pressure. Given the ecology and seasonal anthropogenic pressure impacted on the floodplain, it is evident that human livelihood is dependent on the floodplain which is endemically contaminated with spores. Hence, in the absence of systematic disease intervention programmes the recurrence of anthrax has become a cyclical event that is highly correlated with human activities on the floodplains. This study has revealed that quality veterinary services are critical in preventing anthrax outbreaks and therefore highly recommends improvement in veterinary and livestock extension services delivery and establishment of government anthrax surveillance programme for early detection, vaccination and decontamination of anthrax graves within the upper Zambezi basin.

## Source of funding

Funding under this research was partly through the Centre de Coopération Internationale en Recherche Agronomique pour le Développement (CIRAD) – reference number CC#4 as well as the Southern African Centre for Infectious Diseases Surveillance (SACIDS) – reference number WT087546MA. The authors thank the Ministère Français des Affaires Etrangères for supporting the research through the FSP‐RenCaRe project (FSP no2011/36) and SACIDS. This work was conducted within the framework of the Research Platform ‘Production and Conservation in Partnership’ (http://www.rp-pcp.org).

## Conflicts of interest

The authors declare that there is no conflict of interest.

## Ethics statement

This study was cleared by the University of Zambia board of graduate studies committee and it is part of my Master of Science in One Health Analytical Epidemiology work. All ethical guidelines were followed according to the University of Zambia graduate studies committee. No invasive procedure was done or animals being restrained to get any samples.
